# Supplementary material for: Are carbon emissions trading and green financial instruments synergistic? -Comprehensive quantitative research based on content analysis
Source: PLoS One. 2024 Mar 7;19(3):e0298601. doi: 10.1371/journal.pone.0298601 (PMC10919601; doi:10.1371/journal.pone.0298601)
Supplement: S1 Data — (ZIP) [file pone.0298601.s001.zip › Data/地级市Z_F工作报告.zip!/╕╩╦α/╟∞╤⌠╩╨/└┤╫╘╣1⁄2╓┌║┼í╛┬φ┐╦╩2╛▌═°í┐_2017.docx]

各位代表：

　　现在，我代表市人民政府向大会作工作报告，请予审议，并请各位政协委员和列席人员提出意见。

　　本届政府工作回顾

　　本届政府任期的五年，是全面完成“十二五”目标任务，启动实施“十三五”发展规划的重要时期。五年来，面对复杂多变的宏观经济形势和艰巨繁重的改革发展任务，在省委省政府和市委的正确领导下，全市上下深入贯彻党的十八大和十八届三中、四中、五中、六中全会精神，认真落实习近平总书记系列重要讲话精神，主动适应经济发展新常态，全力稳增长、促改革、调结构、惠民生、防风险，全市经济稳步增长，民生持续改善，社会安定和谐，谱写了庆阳发展史上的新篇章。

　　始终把加快发展作为第一要务，着力扩总量、稳增长，综合实力大幅提升。全市生产总值先后跨上500亿元、600亿元台阶，年均增长11.6%，经济总量居全省第二位。预计今年地方固定资产投资总额达到1190亿元，年均增长18.1%；社会消费品零售总额222亿元，年均增长14.6%；财政总收入135亿元，一般公共预算收入41亿元；城镇居民人均可支配收入25770元，年均增长12.4%；农村居民人均可支配收入7850元，年均增长16.4%。银行业金融机构存款余额871亿元，年均增长16%；贷款余额636亿元，年均增长29%。

　　始终把脱贫攻坚作为一号工程，着力夯基础、促增收，小康建设步伐加快。抢抓国家支持革命老区发展的政策机遇，深入贯彻落实省委省政府“1236”扶贫攻坚行动，全面实施“1+20”精准扶贫精准脱贫工作方案，打响了以通村道路为牵引的基础设施建设和以农民“五变”为内容的富民产业培育“两大扶贫攻坚战”。全市农村公路里程达到9096公里，是2011年的20倍，农村群众出行条件显著改善。建成农村安全饮水工程6.37万处，解决了93.3万群众的安全饮水问题。改造低电压村（组）1030个、农村危房（窑）11万户，实施易地扶贫搬迁9.2万人。农村居民收入显著提高。预计到今年底，累计减少贫困人口77万人，贫困发生率由2011年的40.6%下降到7.5%。

　　始终把产业开发作为主攻方向，着力促转型、增效益，结构调整成效明显。能源产业持续壮大。全力推进石油、煤炭资源开发，累计生产原油3510万吨，加工原油1583万吨。天然气、煤层气、页岩气勘探开发稳步实施。宁正、沙井子、甜水堡矿区总体规划获得国家发改委批复，刘园子矿井建成投产，累计生产原煤131万吨。核桃峪矿井实现“四井”贯通，新庄矿井“三井”落底。正宁电厂、环县电厂、西峰热电联产项目获得核准。环县南湫、毛井一期风电项目实现并网发电。园区对生产要素和产业项目聚集吸附效应进一步增强，累计入驻企业362户。大力发展非公经济，全市各类中小微企业达到8128户。富民产业蓬勃发展。坚持用工业化思维谋划推动农业发展，草畜产业创办了“中盛农牧”全产业链养殖加工企业，带动发展标准化规模养殖场1254个，全市畜禽饲养量持续增加。苹果产业大力推广“海升”模式、“西农”技术，建成万亩片带23个，栽植面积达到167万亩，“庆阳苹果”荣获2016全国名优果品和中国最具影响力十大苹果区域公用品牌。瓜菜产业规模不断扩大，种植面积稳定在150万亩。苗林产业迅猛发展，近三年完成苗林培育313万亩。天富亿多元产业融合发展模式，为城郊失地农民就业创业探索出了新路子。农业产业化经营步伐加快，兴办农产品加工、贮藏、营销企业275户，发展农民专业合作社7437个、家庭农场182个。农业机械化程度达到60%以上。第三产业提质增效。推进文化旅游融合发展，建成了南梁红色景区等3个国家4A级旅游景区，累计接待游客2347万人（次），实现旅游收入104亿元。商贸流通体系更趋完善，市区“十大专业市场”、县乡便民市场标准化建设步伐加快，“市有园区、县有中心、乡镇有站、村社有点”的四级电子商务体系基本形成。全市第三产业占比达到38%，比2011年提高了14个百分点。

　　始终把项目建设作为重要支撑，着力补短板、破瓶颈，城乡面貌显著改善。深入实施“3341”项目工程，城乡基础条件发生了历史性变化。交通建设，庆阳机场4C级改扩建工程建成复航，开通客运航线6条，今年旅客吞吐量可达到30万人（次）；西平铁路庆阳段建成投运，银西高铁庆阳段开工建设，青兰高速雷西段、福银高速西长凤段建成通车，结束了庆阳不通铁路、没有高速的历史；建成二级公路4条403公里，实现了县县通二级公路。水利建设，完成扬黄人饮续建一期、环县苦咸水淡化等重点项目，开工建设小盘河、莲花寺、五台山3个水库，马莲河水利枢纽工程进入国家“十三五”重点支持水利项目名录，可研报告上报水利部。城镇建设，建成了市南区雨洪集蓄保塬生态工程等一批重大市政项目，7县县城和重点小城镇整体功能明显提升。全市城镇建成区面积增加46平方公里，城镇化率达到35%，比2011年提高10个百分点。电力建设，新建扩建35千伏以上变电站16座，改造10千伏以下农村电网1万多公里。生态建设，大力实施再造一个子午岭、固沟保塬、土地整治等工程项目，累计造林413万亩，全市森林覆盖率达到28%。建设梯田146万亩，治理流域2037平方公里，治理率达到46%。环境保护得到加强，环境质量持续改善。

　　始终把改革开放作为强市之路，着力促创新、拓空间，发展活力竞相迸发。重点领域改革取得阶段性成效。“放管服”改革深入实施，市级行政审批事项由2011年底的418项减少到173项，减幅59%；“三张清单一张网”全面建立并投入运行，有效激发了市场主体活力。农村集体土地所有权、宅基地和集体建设用地使用权确权登记基本完成，累计流转土地149万亩，流转率达到23%。财政收支管理改革持续深化，国库集中支付、政府采购等改革措施全面落实。积极推进公共资源交易市场化和投融资体制改革，组建了市公共资源交易中心和庆阳能化、文化旅游等8个国有控股集团公司。成功引进甘肃银行、交通银行在我市设立分支机构，庆阳农商银行挂牌成立，农村产业发展资金合作社实现了行政村全覆盖。医药卫生体制改革有序推进，基本药物制度和分级诊疗制度全面建立。对外合作水平进一步提升。主动加强与丝绸之路经济带沿线国家和地区的交流合作，成功举办了第三届、第四届中国（庆阳）农耕文化节。与意大利卡布拉罗拉市、美国埃德蒙市结为友好城市。在印度、白俄罗斯、斯里兰卡等国家成功举办了经贸文化合作项目推介活动。在阿联酋、白俄罗斯设立了产品营销窗口，对外贸易持续扩大。

　　始终把改善民生作为根本目的，着力强保障、兜底线，公共服务不断优化。社会事业全面加强，财政用于民生的支出占到67%。科技应用步伐加快，推广新技术200多项，研发新产品487种，科技对经济增长的贡献率达到52%。教育事业均衡发展，庆阳职业技术学院挂牌成立，“美丽园丁”教育基金项目成功落户；薄弱学校改造计划全面实施，中小学D级危房全面消除；教师队伍建设不断加强，基础教育质量稳步提升，义务教育普及率达到99%。文化事业繁荣发展，市大剧院、传媒业务技术用房项目建设进展良好，城乡文体活动设施更加完善，市、县级地面无线数字覆盖工程全面建成，受益群众超过百万人。文艺创演成果丰硕，《新绣金匾》、《医祖岐伯》等优秀剧目编排上演，《黄土大塬》等20多部专题片在央视播出。卫生医疗条件显著改善，扩迁建市级医院4个，整体迁建县级医疗机构6个，扩建乡镇卫生院93个，实现了贫困村标准化卫生室全覆盖。计划生育工作持续推进，年度人口控制计划全面完成。体育事业健康发展，成功举办了省第八届少数民族运动会、市第十三届运动会等大型体育赛事，竞技体育水平不断提升，全民健身活动深入开展。就业创业工作不断加强，累计招考安置高校毕业生11411人，非公企业对接安置7613人，城镇新增就业22.3万人。社会保障体系更加健全，城乡基本养老、医疗保险参保率达到98%以上；养老服务设施覆盖了70%以上的城镇社区和近一半的行政村。累计发放各类社会救助资金34.5亿元，有效保障了困难群众的基本生活。社会治安综合治理工作不断加强，矛盾纠纷大排查成效显著，安全生产专项整治扎实开展，食品药品、特种设备、产品质量安全监管力度加大，社会大局和谐稳定。普法宣传深入开展，荣获全国“六五”普法先进城市。民族乡村“1+9”发展规划深入实施，对口援藏工作成效显著。双拥共建、国防动员和民兵预备役工作扎实推进，连续两年获得“全国双拥模范城”称号。统计、外事侨务、地震、人防、老龄、档案、妇女儿童、残疾人、红十字会、气象、消防等工作都有新的进步和提高。

　　始终把廉洁高效作为基本要求，着力转作风、提效能，政府建设全面加强。认真落实市委决策部署，自觉接受人大依法监督、政协民主监督和社会各界监督，累计办理人大代表建议435件、政协委员提案1191件。健全完善政府工作规则和重大事项决策程序，政府决策的科学化、民主化、法治化水平明显提升。严格落实中央“八项规定”、国务院“约法三章”、省委“双十条规定”和市委“22条实施细则”，“三公”经费和会议经费支出逐年下降。扎实开展党的群众路线教育实践活动、“三严三实”专题教育和“两学一做”学习教育，从严落实政府系统党风廉政建设主体责任，强化行政监察和审计监督，推进重点领域专项治理，政风行风持续好转。

　　各位代表，2016年，面对经济下行压力加大、原油价格低位徘徊的不利影响，全市上下积极应对，攻坚克难，打出了精准脱贫、产业培育、项目建设、深化改革、民生保障系列“组合拳”，较好地完成了市三届人大六次会议确定的目标任务，实现了“十三五”发展的良好开局。

　　一是脱贫攻坚成效显著。紧盯全市建档立卡贫困村、贫困人口，集中力量实施基础攻坚、易地搬迁、生态建设、产业培育、人才培训工程，新修农村公路1786公里，实施农电改造2761公里，治理低电压村（组）221个，实现了通村油路、三相动力电全覆盖；新建农村安全饮水工程3.1万处，改造危房（窑）2.82万户。金融扶贫力度持续加大，农村产业发展资金合作社累计发放贷款49亿元，脱贫攻坚资金累计达到145亿元。预计全年可实现12.4万贫困人口脱贫。

　　二是产业开发规模扩大。预计全年生产原油780万吨，加工原油345万吨，比上年增加88万吨。瑞华天然气、永欣石化两个能源开发配套产业项目基本建成。甜水堡2号矿井具备联合试运转条件，核桃峪矿井井巷二期工程正在收尾，新庄矿井获得省发改委开工备案手续。庆阳能化集团螺旋焊管、新型电热节能锅炉项目建成投产。富民产业培育进展良好，新栽苹果15万亩，其中矮化密植标准园7万亩；中盛华美肉羊产业化项目启动实施，西峰2万只、镇原3万只种羊繁育场建成投产；种植高原夏菜、特色瓜菜150万亩；完成苗林结合培育108万亩。文化旅游突围突破，庆城药王洞马嵬驿民俗文化村、镇原北石窟寺文化生态旅游区、正宁黄帝文化景区等重点项目快速推进，宁县“印象义渠”莲花池、庆城北欧风情小镇薰衣草庄园等景点成为假日旅游重要目的地。预计全市文化产业增加值完成10亿元，同比增长16%。电子商务快速发展，电商从业人员超过2万人，交易额突破20亿元，增长33%。

　　三是基础建设高效推进。实施500万元以上项目3120个，同比增加244个。甜水堡至罗儿沟圈高速公路项目获得国家发改委批复并开工建设；采取PPP模式，启动实施了华池打扮梁至庆城、南梁至太白、庆阳至平凉3条高速公路项目。城镇建设步伐加快，10060套棚户区改造项目全面开工建设，续建保障性安居工程2800套；市区新建、扩建供热项目8个，增加集中供热面积280万平方米。成功入围全国海绵城市试点市行列，计划总投资47.3亿元，先期16个样板项目进展良好。生态治理得到加强，新建淤地坝13座，新修梯田30万亩，治理水土流失610平方公里。

　　四是改革开放创新实施。行政审批制度改革持续深化，年内取消调整和下放行政审批事项66项，企业建办审批时限由45个工作日压减到5个工作日以内，市本级政府投资项目审批效率提速66%。公立医院改革力度加大，群众就医看病、费用结算更为便捷。“营改增”试点工作全面推行，为企业减税4100万元。商事制度改革扎实推进， “五证合一”启动实施，大众创业、万众创新的氛围日益浓厚，新增市场主体1.2万户。农村综合改革深入推进，实现了市、县、乡三级土地流转服务机构全覆盖。公务用车制度改革顺利推进。成功举办了中国光彩事业庆阳行暨“民企陇上行”、第六届中国中医药发展大会等重大活动，在国内多个城市举办了项目推介会和苹果展销活动，充分利用兰洽会、敦煌文博会等节会平台，主动上门招商，取得丰硕成果。与华为公司成功签署了云计算大数据中心项目建设合作协议。实施500万元以上招商引资项目365个，到位资金802.5亿元，增长12.4%。

　　五是社会事业统筹发展。省列10件、市列11件民生实事办理有序推进，年底前可全部完成。实施中小学校改薄项目616个，改扩建行政村幼儿园793所，北师大庆阳附属学校11个单体建筑主体工程全部封顶。“千台大戏送农村”、“文化进万家”活动深入开展，群众文化活动更加丰富多彩。医疗卫生与养老服务融合发展，成功进入国家医养结合试点市行列。城区公共自行车服务体系建成投用，市民出行更为便捷。扶持2200名高校毕业生到非公经济组织就业，城镇新增就业5万人。安全生产专项整治扎实有效，社会治安综合治理持续加强。

　　各位代表，五年的发展成就来之不易。这是省委省政府和市委正确领导的结果，是市人大、市政协和社会各界鼎力支持的结果，是全市人民同心同德、真抓实干的结果。在此，我谨代表市人民政府，向各位代表和政协委员，向全市各行各业的劳动者，向各民主党派、工商联、无党派人士和人民团体，向离退休老同志，向驻庆部队和中央、省属驻庆单位，向所有关心支持庆阳发展的人士，致以崇高的敬意，表示衷心的感谢！

　　各位代表，回首过去五年的奋斗历程，我们深切地体会到：无论面临多大挑战，都必须坚持科学发展不动摇；无论遇到多大困难，都必须保持凝心聚力干事创业的良好精神风貌；无论处理多么复杂的问题，都必须把人民的福祉放在首位；无论在任何情况下，都必须大力弘扬南梁精神，勇于负责、敢于担当，埋头苦干、奋发有为，勇往直前、开拓创新，努力创造无愧于时代、无愧于人民的业绩。

　　客观分析、冷静思考，全市经济社会发展还存在不少困难和问题，政府工作也存在一定差距和不足。一是经济增长内生动力依然不足。地方工业实力弱小，非公经济比重偏低，第三产业发展缓慢，特别是受能源市场价格波动影响，油煤开发企业投资锐减，致使经济增长趋缓，财政收入难以完成预算任务。二是脱贫攻坚任务依然繁重。目前全市还有17万贫困人口，这些群众大都居住在中北部干旱山区，生产生活条件差，增收难度大，如期实现脱贫摘帽、全面建成小康社会，还需全市上下付出更加艰辛的努力。三是基础设施建设依然滞后。铁路通达里程短，高等级公路覆盖面小，综合交通网络尚不健全；缺水问题突出，水资源瓶颈制约尚未消除；城乡二元特征明显，区域内发展不够均衡。四是干部作风不实的问题依然存在。政府一些工作人员怕担责、不担责，慢作为、不作为，工作缺乏主动性和创造性，抓落实的力度和破解难题的能力还有差距。对这些问题，我们一定认真对待，采取有效措施，着力加以解决。

　　今后五年发展目标和战略重点

　　今后五年，是脱贫攻坚、夯实基础、全面建成小康社会的决胜时期。把握宏观发展趋势，审视我市发展形势，机遇与挑战并存，机遇大于挑战。国家和省上为革命老区“量身定做”的倾斜政策效应叠加释放，为我们今后加快发展提供了难得机遇。只要我们积极利用有利因素，正视困难问题，因势利导、顺势而为，扎实推进各项工作，就一定能够实现经济社会持续、稳定、健康发展，就一定能够使全市人民生活更加富裕、更加幸福！

　　按照市第四次党代会和全市“十三五”发展规划的战略部署，今后五年工作总体要求是：全面贯彻党的十八大和十八届三中、四中、五中、六中全会精神，深入贯彻习近平总书记系列重要讲话精神，践行发展新理念，主动适应新常态，紧盯全面小康总目标，坚持稳中求进总基调，更加注重提高经济发展质量和效益，围绕“五区一极”战略定位，着力推进“三个生态”创建，决战决胜脱贫攻坚，切实加快产业开发，不断夯实基础支撑，全面深化改革开放，有效保障改善民生，努力实现“十大突破”，加快建设经济繁荣、社会和谐、生态优良、人民富裕的幸福美好新庆阳。

　　奋斗目标是：全市经济总量、城乡居民收入较2010年翻一番；地方固定资产投资总额、社会消费品零售总额稳定增长；财政收入、城镇化水平稳步提高；确保按时序实现整体稳定脱贫，确保与全省全国一道全面建成小康社会。

　　战略重点：

　　一是坚定推进脱贫攻坚。着力在基础扶贫、产业扶贫、金融扶贫、科教扶贫、兜底扶贫上创新举措、精准施策，在争取项目资金、改善生产生活条件、增加贫困群众收入、提升公共服务水平上主动作为、抓实见效，推动脱贫攻坚由“强基础”向“提效益”转变，由“集中攻坚”向“巩固提升”转变，夺取脱贫攻坚、全面建成小康社会的决定性胜利。

　　二是坚定推进首位产业。坚持能源产业开发的方向和决心不动摇，加强与能源开发企业总部的沟通衔接，千方百计争取支持，全力以赴推进石油、煤炭项目建设，深度延伸产业链条，开发高附加值产品，推动能源资源由初级加工向精深加工转化。持续加大原油产能建设，努力使原油产量突破1000万吨；推动实施庆阳石化600万吨扩能改造项目，超前谋划1000万吨升级改造项目，实现由单纯燃料型炼厂向炼化一体化转变；加快天然气、煤层气、页岩气勘探开发，实现所有县城管道输气全覆盖。争取宁北、宁西矿区总体规划获得国家发改委批复，实现刘园子、甜水堡2号矿井达产达标，建成核桃峪、新庄矿井，做好毛家川、九龙川、钱阳山矿井项目前期工作，力争煤炭产能达到6000万吨。谋划发展煤制烯烃等新型煤化工产业。加快推进正宁、环县、西峰热电联产等电厂项目建设，力争陇东特高压直流外送工程审批立项、开工建设，积极发展风力发电、光伏发电等新能源产业。

　　三是坚定推进基础建设。坚持发展抓项目不动摇，统筹推进交通、水利、生态、城镇等基础设施建设。围绕构建立体交通网络，建成银西高铁庆阳段、庆阳至平凉铁路和甜水堡至罗儿沟圈、打扮梁至庆城、南梁至太白、庆阳至平凉高速公路；争取实施庆阳机场4D级改造，建成2至3个通用机场。围绕增强水资源保障能力，改造提升市区和县城、重点小城镇供水设施，尽快开工建设马莲河水利枢纽工程和甜水堡调蓄水库，建成董志塬水厂和引黄入庆工程，全力配合推进白龙江调水工程前期工作，加快构建区域协调的水资源配置体系。着力加强生态文明建设，更加注重环境保护，持续改善生态环境，进一步加大“再造一个子午岭”工程实施力度，确保完成苗林结合培育面积700万亩，全市森林覆盖率达到35%；深入推进固沟保塬和资源开发区生态治理工程，有效遏制水土流失，创建国家黄土地质公园，建设生态宜居庆阳。围绕新型城镇化，协调推进海绵城市、管廊城市、智慧城市、文明城市 “四城同创”。加快美丽乡村建设，提高城乡综合服务功能，促进农业转移人口市民化。加快南梁干部学院建设，力争成为国家级干部教育培训基地。

　　四是坚定推进特色产业。围绕创建国家农业可持续发展试验示范区和西北绿色农产品加工贸易区，坚定不移地发展舍饲养殖、优质苹果、设施瓜菜、苗林培育“四大主导产业” ，全力推进农业特色产业扩规模、上层次、占市场。用足用活国家实施土地“三权”改革、促进农业产业化等相关政策，鼓励工商资本、金融资本投资农业，推动养殖业转变发展方式，种植业调整优化结构，加快建设绿色无公害农产品基地，大力发展循环农业及农产品精深加工，发展壮大龙头企业，促进标准化生产、品牌化经营，形成龙头带基地、基地连农户、产加销一条龙、农工贸一体化的产业化发展格局。加快发展农村二、三产业，引导农民就地开展工副业生产，外出扩大劳务收入，分享工业化、城镇化的成果。用足用好证监会为贫困地区企业上市开通“绿色通道”的政策，积极培育中盛、中庆、能化集团等有实力的企业尽早上市，吸引外地有意愿的企业在我市注册上市。

　　五是坚定推进新兴产业。深度挖掘悠久灿烂的历史文化资源，积极推进南梁红色旅游景区和周祖农耕文化产业园创建国家5A级景区，加快民俗文化产业园和旅游景区景点建设，完善功能配套，联通旅游线路，打造文化品牌，促使文化旅游业繁荣发展，力争文化旅游业产值占到GDP的8%以上，吸纳带动就业20万人以上。积极培育壮大现代物流、健康养老、互联网+、大数据、云计算等新业态，落实好与华为公司签订的战略合作协议，努力把我市打造成为区域大数据处理中心、电商服务中心、现代服务业支撑中心和全省信息消费中心。

　　六是坚定推进民生事业。坚持把保障改善民生作为工作的出发点和落脚点，妥善解决困难群众生产生活问题，下功夫为群众办一批看得见、摸得着的实事好事。加快科技、教育、文化、卫生等各项社会事业发展，着力提升社会保障精准水平，有效促进公共服务均等化，基本实现学有所教、病有所医、老有所养、劳有所得、困有所助、住有所居、安有所保，持续增强人民群众的幸福感。

　　2017年政府工作任务

　　2017年，是全力脱贫攻坚，实现新一届政府工作良好开局的关键之年。全市经济社会发展的预期目标是：生产总值增长7.5%，规模以上工业增加值增长7.5%，地方固定资产投资总额增长8%以上，社会消费品零售总额增长8%，财政总收入增长7%，一般公共预算收入增长7%，城镇居民人均可支配收入增长9%，农村居民人均可支配收入增长10%。城镇登记失业率、万元GDP能耗、污染物排放总量和环境质量均控制在省上下达的指标之内。

　　重点做好九个方面的工作:

　　（一）脱贫攻坚要在培育产业、增加收入、改造危房（窑）上取得新进展。

　　多元化培育富民产业。按照“塬区苹果、山区草畜、川区瓜菜、宜林荒山和林缘区苗林”的产业发展布局，积极推广“海升”模式、“西农”技术，新栽优质苹果10万亩，改造低产园10万亩。大力推广“中盛”现代畜牧业发展模式，更新种植紫花苜蓿100万亩，新建规模养殖场200处，肉羊、肉牛、生猪、肉鸡饲养量分别达到710万只、84万头、178万头和3850万只。积极发展设施瓜菜和高原夏菜，瓜菜总面积达到150万亩以上。持续发展苗林产业，完成苗林培育100万亩以上。有效保障粮食安全，产量稳定在150万吨以上。积极推行政策性农业保险，加强畜禽疫病防治和检验检疫工作，努力增强农业抗御风险能力。

　　多渠道增加群众收入。坚持把促进农产品品牌建设和市场销售作为富民增收的重要保障，积极搭建农产品外销平台，多形式开展宣传推介，进一步扩大地方特色农产品的市场影响力，支持苹果、肉羊等加工流通企业加快发展，着力增加群众的生产性、经营性收入。加大新型职业农民培训力度，完成农民实用技术培训10万人（次），提高农民自我发展能力；完成劳务输转56.5万人，有效增加群众劳务性、工资性收入。全面落实种粮补贴、退耕还林补助等惠农政策，探索推进农村宅基地使用权、土地经营权改革，盘活现有资产，切实增加群众转移性、财产性收入。

　　多举措改造危房（窑）。在持续推进农村断头路、安全饮水工程等基础设施建设的同时，集中财力物力人力，实施好危房（窑）改造项目。按照政府主导、规划引领、群众参与、突出特色的原则，将农村危改与易地搬迁、美丽乡村建设结合起来，解决1.85万户群众危房（窑）问题，确保实现农村危房（窑）改造全覆盖。

　　（二）首位产业要在争取支持、加快开发、扩大产能上谋求新突破。

　　锲而不舍争取项目实施。进一步加强与中石油集团对接，争取庆阳石化600万吨炼油升级改造项目取得实质性进展；汇报争取国家能源局、国家电网公司支持，尽早实施陇东至华东±800千伏特高压直流外送工程，努力推进首位产业开发核心项目取得新突破。

　　推动油田加快产能建设。全力支持长庆油田扩能增产，争取中石化华北油田恢复启动境内油田生产，促使新增石油探明储量2亿吨、原油产量达到800万吨；全力支持庆阳石化满负荷生产，原油加工量达到365万吨。积极延伸石化产业链条，支持瑞华天然气、永欣石化两个配套产业项目投产运行。

　　持续推进煤电开发建设。围绕煤炭资源开发转化，促使刘园子矿井达产达标，争取甜水堡2号矿井开展联合试生产，协调实施好核桃峪、新庄等矿井建设，开工建设马福川矿井；力争开工建设正宁电厂、环县电厂，确保西峰热电联产项目全面开工；加快新能源建设步伐，力争环县、华池、西峰、镇原集中式光伏电站和环县毛井二期等风电项目建成并网发电。

　　（三）基础建设要在统筹谋划、上下联动、加大投资上再下新功夫。

　　抓谋划争取。紧盯《陕甘宁革命老区振兴规划》、《陇东能源基地开发规划》等政策利好，深度研究，逐条对接，在基础设施建设、优势资源开发、特色产业培育、文化旅游发展、生态环境保护、民生保障改善等方面，精心谋划储备一批大项目、好项目，论证实施一批PPP项目。加强与国家有关部委、省直相关部门的衔接争取，确保我市有更多的项目进入国家和省上计划“盘子”。

　　抓开工建设。坚持大、中、小项目齐抓，民生类、产业类、基础类项目并重，力争南梁通用机场、庆阳至平凉铁路、马莲河水利枢纽工程等重点项目开工建设；加快银西高铁庆阳段和甜水堡至罗儿沟圈、打扮梁至庆城、南梁至太白、庆阳至平凉高速公路建设，实施好庆城至周祖陵景区一级公路和新庄湾至桐川、杨新庄至南梁、殿王家至平泉、阳路湾至席家沟圈、五里墩至盘克等省道公路项目；推进扬黄人饮向庆城延伸供水工程，完成五台山、小盘河水库主体工程，加快莲花寺水库和董志塬水厂建设，力争开工实施甜水堡调蓄水库，提高水资源保障能力。

　　抓投资进度。加强项目资金调度，积极盘活存量，充分用好增量，高效管理使用，切实保障项目建设的资金需求。健全完善主体责任清晰、进度时限明确、制约措施严格的项目投资落实机制，继续实行“三个一”包抓责任制，确保各类重大项目按计划实施、快速度推进、高质量建设，推动形成更多的投资实物量，以投资扩总量、促发展。

　　（四）第三产业要在突围突破、大力扶持、培育壮大上打造新亮点。

　　做大做强文化旅游业。把文化旅游产业作为促进第三产业增长的突破口和发力点，制定出台文化旅游产业发展的政策措施，在规划引领、资金投入、配套设施建设等方面加大支持力度。持续推进民俗文化产业园、北石窟寺文化生态旅游区、马嵬驿民俗文化村等重点项目建设，完善景区道路、停车场等配套服务设施，提升整体服务功能。统筹市内旅游资源，开发生态观光、中医养生、文化寻根、休闲度假等多样化旅游产品，推进“资源互享、优势互补、游客互送”，促进旅游产业由“零散化、雷同化”向“协同化、差异化”方向转变。更加鲜明地“举革命老区旗、走红色旅游路”，加强与西安、银川、兰州等周边地区旅游线路的对接，联合打造“会宁-六盘山-南梁-延安”红色旅游精品线路，增强我市旅游品牌的核心竞争力。

　　改造提升传统服务业。加快交通运输、商贸流通、住宿餐饮等传统业态改造升级，引导相关行业主体提升质量、优化服务，推动生产型服务业向专业化和价值链高端延伸，促进生活型服务业向精细化和高品质方向转变。引导扩大居民住房、汽车、旅游等消费，培育新的消费热点，拉动经济增长。

　　积极发展现代服务业。进一步促进信息技术、金融保险、生物医药、养老服务、现代物流等新兴产业加快发展。尽快开工建设华为云计算大数据中心项目，谋划建设“双创”信息产业园。全力实施第二批电信普遍服务建设项目，促进行政村宽带全覆盖。依托市电子商务创业孵化园，加快电商公共服务平台建设，完善服务要素，推动线上线下融合发展。

　　（五）生态建设要在工程推进、机制完善、依法治理上再创新业绩。

　　把生态文明建设摆在更加突出的位置，牢固树立绿色发展、循环发展、低碳发展理念，持续抓好“再造一个子午岭”、固沟保塬和资源开发区生态治理“三大工程”建设，强化环境保护和生态修复，节约集约利用土地、水、能源等资源，形成人与自然和谐发展新格局。

　　持续推进再造子午岭工程。围绕创建国家生态安全屏障综合试验区，扎实推进天保二期、三北五期、退耕还林还草等重点生态工程，加快荒山、道路、村镇、城区和油田矿区绿化，建设绿色庆阳。

　　持续推进固沟保塬工程。组织实施好5个国家黄土塬面保护项目，扎实推进坡改梯和重点水保续建工程，完成40个淤地坝除险加固工程，治理水土流失600平方公里，其中新修梯田20万亩、小型水保拦蓄工程1900座，有效控制塬面萎缩。

　　持续推进生态环境治理。贯彻市人大即将颁布的《庆阳市禁牧条例》，落实禁牧措施，保护林草植被，巩固造林绿化成果；发展循环经济，节约能源资源，严格水源地保护，促进节水型社会建设；健全地企共建共担的生态保护与修复责任机制，加大油区、矿区等重点区域综合治理；全面落实国务院“大气十条”、“水十条”、“土十条”等环境保护政策法规，着力推进大气、水体、土壤污染和地质灾害防治，加大环境监管执法力度，减少污染物排放，切实改善环境质量。

　　（六）城镇建设要在规划引领、建管并重、功能完善上强化新举措。

　　突出规划引领。加快城市总体规划修编暨“多规合一”编制工作，做好镇、乡控制性详细规划编制，突出地域特色和文化内涵，塑造城市个性，展现城市风貌。更加注重城市空间规划布局的科学性和前瞻性。严格规划管理，强化规划的刚性约束。

　　突出功能提升。积极做好全国管廊城市建设试点争取工作，实施好市区重点路段、住宅小区、广场绿地、水系湖体的海绵化改造，加快市区供水供热供气管网、公交站点、南部生态绿地等市政设施建设，完成棚户区改造10832户。加大县城开发和特色小镇、美丽乡村建设力度，使之成为宜居、宜业、宜游之地，成为带动发展之源。

　　突出精细管理。积极推进全国文明城市创建，在市区、县城实施硬化、绿化、亮化、净化、美化“五化工程”，持续加大主次干道、背街小巷、公共场所、城乡结合部等重点区域部位环境综合整治力度，着力解决城市脏乱差和交通拥堵等突出问题，坚决查处随意燃放烟花爆竹等不文明行为。强化市民养成教育，努力提升文明素质。以智慧教育、智慧医疗、智慧交通、智慧政务为切入点，推进城市管理信息化，建设智慧城市。强化城镇社区服务能力，规范住宅小区物业管理，加强房地产市场监管，提高治理城市、服务市民的综合水平。

　　（七）改革开放要在破解难题、优化环境、合作共赢上展示新气象。

　　聚焦重点领域，破解发展难题。按照中央和省委、市委的决策部署，全力推进重点领域改革，有效破解影响发展的体制机制性障碍。深化农村综合改革，引导土地经营权规范有序流转。加快推进医药卫生体制改革，促进新农合、城镇居民医保、干部职工医保“三保合一”，落实药品零差价和分级诊疗制度。深化财政管理制度改革，积极推进政府购买社会服务，加强国有资产管理，提升资金资产使用效益。持续推进市区公交运营体制改革，科学设置线路，方便市民出行。健全农村公路养护机制，提高农村公路管理水平。深化投融资体制改革，完善地方金融体系，争取引进浦发银行、光大银行、中信银行在我市设立分支机构，巩固农村产业发展资金合作社试点成果，持续扩大信贷规模，发展普惠金融，增强金融支持地方发展的能力；加强社会信用体系建设，改善金融生态环境，有效防范金融风险。

　　优化投资环境，全力扶持民营经济。牢固树立“环境就是生产力”的理念，进一步简化项目审批程序，实行 “一站式”办理，提供“一条龙”服务。落实招商引资优惠政策，完善土地供应、基础配套等保障要素，提高土地利用和投资强度，着力解决效率不高、诚信不足、环境不佳的问题，营造亲商安商的人文环境、高效廉洁的政务环境、文明规范的法治环境、公平公正的社会环境，构建“亲”“清”的政商关系，努力让企业在我市放心投资、放手发展。积极支持民间资本投资实体经济，引导企业淘汰落后生产工艺和技术设备，加快产品升级换代，帮助企业引进、培养技术管理人才，建立现代企业制度，走创新发展之路。认真落实“互惠贷”、“助保贷”、“互助贷”等扶持政策，有效破解民营企业发展资金短缺问题。

　　树立全球视野，扩大对外开放。积极融入“一带一路”，加强与中西亚、中东欧国家的经贸往来；深化与关天经济区、西咸新区、宁蒙陕甘毗邻地区和长三角、京津冀等发达地区的经济合作。充分利用敦煌文博会、兰洽会、广交会、农耕文化节等节会平台，大力发展会展经济，促进我市特色农产品、民俗文化产品对外销售，实现区域、企业、产业融合发展。坚持“走出去”与“引进来”相结合，着力在引新、引高、引强上求突破，力争更多有实力的企业落户我市，切实提高招商引资合同履约率、资金到位率和落地开工率。

　　（八）平安建设要在综合施策、标本兼治、长治久安上实现新变化。

　　加强和创新社会治理。健全完善立体化社会治安防控体系和应急处置机制，加强各类风险防控，提高应对突发事件的能力。推进公共安全视频监控联网应用项目建设，整合监控资源，提升社会面治安防控水平。加大对“盗抢骗”和严重暴力犯罪的打击力度，促使全市刑事发案率明显下降，切实增强人民群众的安全感。强化“七五”普法宣传教育，构建覆盖城乡的公共法律服务体系。坚持领导干部接访下访制度，畅通信访渠道，努力把矛盾纠纷消除在源头。

　　强化安全生产和公共安全监管。严格落实安全生产责任制，突出抓好道路交通特别是农村道路交通安全，紧盯重点区域和事故高发路段，规范标志标线，完善安全设施，严查严治违章违规行为，有效遏制道路交通事故频发多发势头。持续抓好油气管道、危险化学品、消防、煤矿和非煤矿山、建筑工地等重点领域安全监管，加强校园、商场、车站等人员密集场所安全管控，促进防范措施落实到位、各类隐患排查整改到位，全力保障人民群众生命财产安全。加大食品药品监管力度，让人民群众吃上放心食品、用上安全药品。

　　（九）民生事业要在全民共享、机会均等、和谐发展上取得新成效。

　　全面推进社会事业发展。着力培育科技创新主体，推广政产学研用合作模式，积极推进国家农业科技示范园区和科技大市场建设，促进科技成果转化应用。继续实施义务教育学校标准化建设工程，实施普通高中教育攻坚计划，大力发展学前教育、职业教育和特殊教育，促进教育均衡发展；完成北师大庆阳附属学校建设，确保2017年秋季招生；加快城区中小学校和幼儿园建设，切实解决入学难的问题。加强城乡公共文化设施建设，建成市大剧院、传媒业务技术用房等重点工程，启动市文化馆、图书馆和画院建设，广泛开展群众性文化活动，有效满足群众文化生活需求。深入推进全民健身活动，积极备战全省第十四届运动会。加强医疗机构基础能力建设，启动市人民医院扩容提能工程前期工作，加快市妇女儿童医院和县、乡医疗卫生机构改扩建；加强卫生人才队伍建设，提升医疗服务水平，构建和谐医患关系；深入推进“健康庆阳”建设，提升群众健康水平。积极扶持引导高校毕业生到基层、非公企业就业，做好复转军人、困难家庭和零就业家庭就业帮扶工作，促进创业带动就业；实施全民参保计划和机关事业单位养老保险制度，提高城乡低保标准，扩大住房公积金覆盖范围，积极发展社会福利和残疾人、慈善事业，关爱农村留守儿童，创新社会救助机制，健全城乡养老服务体系，筑牢保障困难群众基本生活的防线。做好民族宗教工作，进一步改善少数民族乡村基础设施条件。

　　扎实办好民生实事。在认真落实惠民政策、完成省上民生实事办理的同时，安排实施10项市列民生实事：（1）解决10.4万农村群众安全饮水问题；（2）培训农村学校紧缺薄弱学科教师330名；（3）实施10所中小学“智慧校园”建设项目；（4）救助高考入学困难家庭学生；（5）培训医疗卫生技术人员334名；（6）建设“乡村舞台”135个；（7）实施市区48条主次干道、42条巷道综合治理工程；（8）继续实施市区公共自行车建设项目，新增服务网点36个、投放自行车1000辆；（9）修建环卫工人休息室及文明公厕20处；（10）实施市区老旧供热管网提质改造工程。

　　关于加强政府自身建设

　　打铁还需自身硬。加强政府自身建设是做好政府工作的前提和基础。面对加快发展的重任和全市人民的期盼，新一届政府将更加重视自身建设，集聚精气神，全力促发展，做到为人民服务、对人民负责、受人民监督、让人民满意。

　　第一，建设法治政府，推进依法行政。坚决落实中央和省委省政府各项决策部署，执行市委决定，接受人大依法监督、政协民主监督，接受司法、舆论和社会监督，认真办好人大代表建议和政协委员提案。深入学习《宪法》、《行政诉讼法》等法律法规，全面贯彻《法治政府建设实施纲要》（2015-2020年），加强政府立法工作，推进依法行政、依法办事，切实提高运用法治思维和法治方式推动工作、解决问题的能力。坚持公众参与、专家论证、风险评估、合法性审查和集体讨论决定制度，广泛听取民主党派、工商联、人民团体和各界人士的意见建议，切实保障群众的知情权和参与权，提高政府工作的透明度。

　　第二，践行群众路线，推进为民行政。始终把人民群众放在心中最高位置，把面向群众、为人民服务作为政府工作人员的根本遵循，大力弘扬“南梁精神”，坚持深入群众、依靠群众，问政于民、问计于民、问需于民，保持与人民群众的血肉联系。善于站在群众的立场想问题，带着对群众的深厚感情做工作，确保把更多时间和精力用在帮助群众解决实际困难上，把更多财力和物力用在提高人民生活水平上，让发展成果更多、更广泛地惠及广大人民群众。

　　第三，着力改进作风，推进务实行政。大兴求真务实之风，加强调查研究，深入基层、深入一线，听实话、察实情、出实招，做到谋划工作实、推动发展实、解决问题实、措施保障实。大兴开拓创新之风，敢于直面矛盾，勇于解决问题，在创新中提升能力，在实干中开创新局。大力弘扬“钉钉子”精神，强化责任担当，推进各项部署落细落小、落地落实，坚决反对做表面文章、搞形式主义，坚决防止弄虚作假、虚报浮夸，坚决杜绝形象工程、政绩工程，让担当实干成为政府工作人员的自觉追求。

　　第四，健全运行机制，推进高效行政。深入推进简政放权、放管结合、优化服务，建立完善权力清单、责任清单和负面清单，大力推行网上审批服务和限时办结制度，扩大政务公开，提高行政效能。强化目标管理，层层分解任务，一级抓一级，一级对一级负责。加强上下联动、团结协作，确保政令畅通、执行有力。严格督查考核，强化问责问效，坚决克服懒政、怠政，坚决惩处失职、渎职行为，确保各项工作高标准推进、高效率完成。

　　第五，严格监督约束，推进廉洁行政。深入贯彻党的十八届六中全会精神，落实全面从严治党要求，严格执行《关于新形势下党内政治生活的若干准则》和《中国共产党党内监督条例》，严守政治纪律和政治规矩，切实提高政府机关和工作人员自我净化、自我完善、自我约束的能力。驰而不息反对“四风”，坚决执行中央和省、市委反腐倡廉各项规定，认真贯彻省委“3783”党风廉政建设主体责任体系、“866”衡量检验标尺和“111”督查落实细则，切实履行“一岗双责”，层层传导压力，倒逼责任落实，把严的标准和实的要求贯穿于政府工作的全过程。加强政府工作人员教育管理，坚持用制度管权、管事、管人，强化行政监察和审计监督，严肃查处违法违纪案件，坚决防止和纠正损害群众利益的不正之风。大力发扬艰苦奋斗的优良传统，厉行节约，反对浪费，严格财经纪律，规范公务行为，弘扬清风正气。

　　各位代表，脱贫攻坚重任在肩，全面小康催人奋进。我们要在省委省政府和市委的正确领导下，在市人大、市政协的监督支持下，充分激发调动全市广大干部群众的积极性，同心同德，开拓创新，扎实工作，确保圆满完成各项目标任务，为建设幸福美好新庆阳而努力奋斗!
